# Factors associated with health services financier among temporary sheltered homeless in urban Malaysia

**DOI:** 10.1186/s12889-019-6871-5

**Published:** 2019-06-13

**Authors:** Azimatun Noor Aizuddin, Siti Waffa Abdul Jabar, Idayu Badila Idris

**Affiliations:** Department of Community Health, Faculty of Medicine, Universiti Kebangsaan Malaysia, Jalan Yaacob Latif, 56000 Bandar Tun Razak, Cheras, Kuala Lumpur Malaysia

**Keywords:** Homeless, Health utilization, Health financing, Health service, Health financier

## Abstract

**Background:**

The presence of homelessness in Malaysia is not a new issue. The existence of homeless population is growing, along with the development of this country. With the increasing number of homelessness, the range of issues, such as health services financier among them, has surfaced. However, there was limited study conducted on this subject. The main objective of this study was thus, to identify the financier of health services among the homelessness in Kuala Lumpur and factors associated with it.

**Methods:**

In this cross-sectional study, we include 196 homeless people aged above 18 years, Malaysian who were able to communicate with interviewers, and respondents who were not aggressive. These respondents were transits at Pusat Transit Gelandangan Kuala Lumpur and Anjung Singgah Kuala Lumpur and were available during interview sessions. They were selected via simple random sampling and were interviewed via face to face guided interviews using a validated structured questionnaire. Data were analysed descriptively, as well as using bivariate and multivariate analysis to explore the associated factors.

**Results:**

The study showed that 57.7% homeless utilized the health services with only 37.8% assessed government health services. Only 42.5% of the respondents use their own money and 46.9% received aids to finance their health. Major influencing factors that influence homeless people to use their own money for health services were education level, income and disability, with adjusted OR (95% CI) of 3.15 (1.07–9.25), 0.08 (0.029–3.07) and 0.05 (0.003–0.88) while *p* value was 0.037, < 0.001 and 0.041 respectively. The influencing factors for receiving aid for health services were income and those who took drugs with adjusted OR (95% CI) of 6.50 (2.30–18.39), and 0.33 (0.11–0.95) while *p* value was < 0.001 and 0.041 respectively.

**Conclusion:**

There is low healthcare services utilization and affordability among homelessness. All parties should play a role in ensuring that homeless people are not left behind in the health care accessibility in Malaysia.

## Background

The presence of homelessness in Malaysia is not a new issue. The existence of homeless population has been on the rise, along with the development of this country. Kuala Lumpur City Council (DBKL) recently reported that at least one to two thousands homeless people were at the Kuala Lumpur City Center [1]. The existence of these homeless people is not only seen in Southeast Asia such as Malaysia and Indonesia but many more were found in neighboring countries. Their presence was also documented in the European countries. Based on reports released by the United Nations High Commissioner on Refugee (UNHCR), there are at least 100 million homeless people around the world [2]. From UNHCR record in 2005, there was 15,000 people who were homeless in Argentina. In the United States of America (USA) the number rises to 600,000 to 2.5 million people. Canada recorded the number of homeless people as 9, 465 and more [2]. This clearly shows that, homeless people is indeed a global concern [2].

Currently, the homelessness daily needs such as food, shelter and wellness treatment were provided by non-governmental organizations (NGOs). In Malaysia, most NGOs, such as the Soup Kitchen, emphasizes on feeding alone [3]. To date, only the Yayasan Kebajikan Negara (YKN) focuses on other aspects including accommodation, medicine and job provider for the homeless community, with the establishment of the Anjung Singgah in Kuala Lumpur [3]. However, the services offered are limited. These homeless people require help from other sources, as most of them have no income or having very low income. According to Rafiza, at least 17.7% of the homeless have zero income [4]. Jobs and income also known to be related with education level. Homelessness in the Malaysian Policy Sheet reported that 56% of homeless people in Malaysia have primary education only [5]. Additionally, according to Shelton et al., there were several risk factors that cause homeless people to have no education or have low education [6]. This is due to socioeconomic problems such as not having the opportunity to spend on education because of financial problems [6].

According to the Fourth Edition Dewan Bahasa dan Pustaka Dictionary, homeless person or *gelandangan* in Malay means a person who does not have a permanent job or residence [7]. National Coalition of the Homeless also supports the definition of homelessness in which a homeless person is defined as an individual who does not have a fix and comfortable place to stay especially at night [8]. Similarly, in their brief policy report, Shelter England defines the homelessness as a group of people who do not have homes [9]. On the other hand, Canadian Homelessness Research Hub defined homelessness as persons who are faced with a situation where they and their families do not have a stable life, not having the right house and not being able to have it [10]. The National Coalition of the Homeless also added that this situation occur due to the improvement of living standards where many individuals have difficulty in getting a home within an affordable price [8]. According to Datuk Dr. Denison in Star Metro, one of the main contributing factors to Kuala Lumpur’s homelessness problem is the lack of affordable housing for the poor [11]. The homeless population is therefore considered as a community group that is often viewed cynically. This group is well known as street people or street friends [3].

According to Mohamad et al., homeless population are among the group of people that are often dropped out or missed from receiving any help due to the absence of any specific guidelines or policies that specify their needs and destiny [12]. According to Homeless in the Malaysian Policy Sheet, homeless people are usually consisted of the elderly men who are single and jobless [5]. With the increasing number of homeless populations, various issues that are related to them has surfaced. Among essential issue is health inequalities. Few volunteers among health professionals conduct health screening and offer basic treatment for free to the homeless people, although this is not on regular basis. We are yet to get answers on few questions such as follows. Do homeless people have diseases? What diseases do they have? Do they get treatment for their diseases? How and where do they get their treatment? How do they pay for their treatment or who finance their treatment? And finally, what factors associate with their health services financier?

It was found by previous studies that one of barriers to health services among homeless is lack of insurance [13] and lack of ability to pay for health services [14]. According to Adam and Ke Xu, inpatient expenses is the factor associated with borrowing and depleting assets for health services [15].

There are few studies conducted among homeless people in Malaysia and very limited conducted on health-related issues especially on health services financier. The main objective of this study was to identify the financier of health services among the temporary sheltered homelessness in Kuala Lumpur and the factors associated with it. This study findings will suggest to policy makers to take an appropriate action and focus more on specific support in health services deliveries to the homeless population with collaboration of NGOs.

## Methods

A cross-sectional study was conducted among homeless people who temporarily transit at Pusat Transit Gelandangan Kuala Lumpur and Anjung Singgah Kuala Lumpur from January till March 2018. Both temporary shelter homes for the homelessness were almost similar in nature. There were almost 400 homeless people who transit at both places at any one time. To answer the study objectives, sampe size was calculated based on previous studies which were used as references. The calculated sample size was 196, and an additional 10% were included considering the non-respondents. The final total minimal sample needed for the study was 216 considering that not all homeless people would be available during survey period and interview sessions.

The respondents were randomly selected according to the inclusion criteria, i.e. those with the age of above 18 years, Malaysian and able to communicate with the interviewer. Respondents who were aggressive and who were not in during interview sessions, were not included in this study. Individuals were face to face interviewed using questionnaire that was partially adopted from Aizuddin et al. and modified accordingly, to suite the homelessness as study population [16]. The questionnaire was assessed for content validity by the experts and face validity was evaluated during the pretest. The questionnaire was divided into four parts namely sociodemographic, socioeconomic, health related and healthcare utilization, as well as a section on health financing.

Individuals were asked on their birth dates, sex, ethnicity and marriage status in the sociodemographic section. Socioeconomic questions consisted of education level, working status and their income. For health related, individuals were asked on questions such as whether they smoked or not, consumed alcohol or not, were drug users or not, whether they had any disease or not and whether they had any disabilities or not. For healthcare utilization, individuals were asked on where do they get treatment for their health problem. For financier part, which constitute the dependent variable, individuals were asked on who paid for their health services, whether they use their own money or otherwise and whether they get any aid to finance their health services.

During the initial phase, data were entered into EXCEL and transferred to IBM SPSS Predictive Analytics software version 20.0 for analysis. All data were first explored descriptively before proceed to bivariate analysis. As all data are categorical, the data were presented in numbers and percentage, and Pearson Chi-square analysis were performed for bivariate analysis. For mulvariate analysis, Multiple Logistic Regression were used. For that purpose, few independent variables were recategories: marital status [single and married (plus others)], working status [working and not working (plus beggars)] and education level (low and middle – high).

The research was reviewed and approved by the Medical Research Ethic Committee, Faculty of Medicine, Universiti Kebangsaan Malaysia with Ethic Number FF-2018-019. All participants also provided informed consent before interviews commenced.

## Results

A total of 196 individuals were interviewed, giving a respond rate of 90.7%. Majority of the respondents were male (87.8%) with mean age of 42.5 and standard deviation (SD) of 10.1. More than half were single (63.8%). This study has shown that 70.9% of respondents obtained education till form five, 95 (48.5%) of respondents were working and 88 (49.9%) of them had some form of income with mean and SD of RM766.4 ± 293.0 respectively. Around 74.0% of respondents were smokers, 84.7% did not consume alcohol and 66.8% respondents had history of drugs addiction. Result also showed that 57.7% of respondents had disease with 53.0% of them had chronic diseases while 16.8% were Human Immunodeficiency Virus (HIV) positive. Half of the respondents (57.5%) utilized health services with only 37.8% used government health services. In terms of financing their health, only 42.5% of respondents used their own money while 46.9% received aid from others.

The association between independent factors and using own money for health services among homeless people is shown in Table 1. For socio-demography factors, it is shown that respondents who used their own money for health services were higher among male, married and who were in their middle to late adulthood age compared to female, single and respondents who were in their early adulthood age. However these differences were not statistically significant. For socio-economic factors, it is shown that there were significant association between working status and income with using own money for health services. Proportion of respondents who used their own money for health services were higher among working respondents compared to non-working respondents and respondents who were beggars. Proportion of respondents who used their own money for health services were higher among respondents with no income compared to respondents with income. For health related factors, it is shown that respondents who smoked, did not drink alcohol, did not take drugs, had diseases and those with disabilities showed higher percentage in using their own money for health services compared to those who did not smoke, drink alcohol, took drugs, did not have diseases and those without disabilities. However, all these associations were not statistically significant.

**Table 1:** Bivariate analysis of using own money as health services financier (*n* = 113)

Variables	Own money n (%)	Not own money n (%)	*ϰ* ^2^ value	p value
Gender				
Male	42 (44.7)	52 (55.3)	1.110	0.292
Female	6 (31.6)	13 (68.4)		
Age				
Early adulthood	22 (41.5)	31 (58.5)	1.979	0.372
Middle-Late adulthood	26 (47.2)	29 (52.8)		
Marital Status				
Single	30 (42.3)	41 (57.7)	0.278	0.870
Married	13 (40.6)	19 (59.4)		
Others (Widow/Widower)	5 (50.0)	5 (50.0)		
Working Status				
Working	28 (53.8)	24 (46.2)	9.358	0.009*
Not working	18 (40.9)	26 (59.1)		
Beggars	2 (11.8)	15 (88.2)		
Income				
With	22 (28.2)	56 (71.8)	20.995	< 0.001*
Without	26 (74.3)	9 (25.7)		
Level of Education				
Low	16 (50.0)	16 (50.0)	1.4.34	0.488
Middle	28 (38.4)	45 (61.6)		
High	4 (50.0)	4 (50.0)		
Smoking				
Yes	35 (43.8)	45 (56.2)	0.181	0.670
No	13 (39.4)	20 (60.6)		
Took Alcohol				
Yes	3 (23.1)	10 (76.9)	2.263	0.133
No	45 (45.0)	55 (55.0)		
Took Drugs				
Yes	14 (41.2)	20 (58.8)	0.034	0.854
No	34 (43.0)	45 (57.0)		
Disease				
Have	29 (40.8)	42 (59.2)	0.208	0.648
No	19 (45.2)	23 (54.8)		
Disabilities				
Have	1 (16.7)	5 (83.3)	1.728	0.189
No	47 (43.9)	60 (56.1)		

* Significant at *p* < 0.05

Table 2 shows the association between all independent variables and the dependent factor, which was whether respondents received aid for health services. For socio-demographic factors, it is shown that there was a significant association between gender and received aid for health services, in which more female received aid for health services compared to male. Apart from that, more middle to late adulthood and married respondents received aid for health services compared to early adulthood and single respondents but these differences were not statistically significant. For socio-economic factors, it is shown that only income status showed significant association with receiving aid for health services. The proportion of respondents who received aid for health services was highest among those who have income as oppose to those who did not have income. It is also shown that highest proportion of those receiving aid were among beggars and respondents with low education level compared to those who worked or did not work and those who had middle or high education level. Nevertheless, these associations were not statistically significant. For health related factors, it is shown that only taking drugs had a significant association with received aid for health services. Proportion of respondents who did not take drugs were higher to receive aid for health services as compared to those who were drug users. Respondents, who did not smoke, did not take alcohol, did not have disease and did not have disabilities had higher percentage of receiving aid for health services as oppose with those who smoked, consumed alcohol, had disease and had disabilities. However these associations were not statistically significant.

**Table 2:** Bivariate analysis of received aid as health services financier (*n* = 113)

	With Aid n (%)	Without Aid n (%)	*ϰ* ^2^ value	p value
Gender				
Male	40 (42.6)	54 (57.4)	4.247	0.039*
Female	13 (68.4)	6 (31.6)		
Age				
Early adulthood	24 (45.3)	29 (54.7)	0.707	0.707
Middle-late Adulthood	29 (48.3.0)	31 (51.7)		
Marital Status				
Single	32 (45.1)	39 (54.9)	0.258	0.879
Married	16 (50.0)	16 (50.0)		
Others (Widow/Widower)	53 (46.9)	60 (53.1)		
Working status				
Working	24 (46.2)	28 (53.8)	5.049	0.080
Not working	17 (38.6)	27 (61.4)		
Beggars	12 (70.6)	5 (29.4)		
Income				
With	46 (59.0)	32 (41.0)	14.736	< 0.001*
Without	7 (20.0)	28 (80.0)		
Level of Education				
Low	16 (50.0)	16 (50.0)	1.696	0.428
Middle	35 (47.9)	38 (52.1)		
High	2 (25.0)	6 (75.0)		
Smoking				
Yes	34 (42.5)	46 (57.5)	2.132	0.144
No	19 (57.6)	14 (42.4)		
Took Alcohol				
Yes	5 (38.5)	8 (61.5)	0.420	0.517
No	48 (48.0)	52 (52.0)		
Took Drugs				
Yes	11 (32.4)	23 (67.6)	4.134	0.042*
No	42 (53.2)	37 (46.8)		
Disease				
Have	30 (42.3)	41 (57.7)	1.658	0.198
No	23 (54.8)	19 (45.2)		
Disabilities				
Have	2 (33.3)	4 (66.7)	0.468	0.494
Non	51 (47.7)	56 (52.3)		

* Significant at *p* < 0.05

The influencing factors for homeless to use their own money for health services were further analysed using multiple logistics regression. The findings are shown in Table 3. All variables were entered into the multivariate analysis. It was shown that the significant variables in the final model were education level, income and disability, with adjusted odd ratio (OR) (95% CI) 3.15 (1.07–9.25), 0.08 (0.029–3.07) and 0.05 (0.003–0.88) respectively and *p* value 0.037, < 0.001 and 0.041 respectively. The Pseudo R-square calculated was 0.352, which indicates that 35.2% of the identified factors influenced the usage of own money for health services in this model. A chi-square statistic was utilized to assess the difference in − 2 log-likelihoods between the final model and a reduced model. It was found that the reduced model is equivalent to the final model because omitting the effect does not increase the degree of freedom.

**Table 3:** Factors associated with using own money for health services among homeless (multiple logistic regression)

Variable	Regression coefficient (b)	Std error	Wald Z value	Adjusted odds ratio (95% CI)	*p*-value
Age					
Early adulthood	0.037	0.466	0.006	1.04 (0.42–2.59)	0.937
Middle-late adulthood	0				
Gender					
Male	0.73	0.742	0.977	2.08 (0.48–8.92)	0.323
Female	0				
Marital status					
Single	−0.140	0.491	0.082	0.87 (0.33–2.27)	0.775
Married	0				
Education level	1.147	0.549	4.361	3.15 (1.07–9.25)	0.037*
Low	0				
Middle-high					
Working status	0.572	0.501	1.301	1.77 (0.66–4.73)	0.254
Working	0				
Not working					
Income					
With	−2.46	0.559	19.416	0.08 (0.029–3.07)	< 0.001*
Without	0				
Smoking					
Yes	−0.13	0.638	0.040	0.88 (0.25–3..07)	0.841
No	0				
Took Alcohol					
Yes	−1.11	0.794	1.956	0.33 (0.69–1.56)	0.162
No	0				
Took drugs					
Yes	0.221	0.543	0.166	1.25 (0.43–3.62)	0.684
No	0				
Disease					
Have	−0.437	0.482	0.820	0.65 (0.25–1.66)	0.365
No	0				
Disability					
Have	−3.012	1.473	4.183	0.05 (0.003–0.88)	0.041*
No	0				

* Significant at *p* < 0.05

Table 4 shows the influencing factors for homeless population who received aid for health services. All variables were entered during the multivariate analysis. It was shown that the significant variables in the final model were income and history of taking drugs with adjusted OR (95% CI) 6.50 (2.30–18.39) and 0.33 (0.11–0.95) and *p* value < 0.001 and 0.041 respectively. The Pseudo R-square calculated was 0.276, which indicates that 27.6% of the identified factors influenced the receiving of aid for health services model in this analysis. A chi-square statistic was further utilized to check the difference in − 2 log-likelihoods between the final model and a reduced model. It was again found that the reduced model is equivalent to the final model because omitting the effect does not increase the degrees of freedom.

**Table 4:** Factors associated with received aid for health services among homeless (Multiple logistic regression)

Variable	Regression coefficient (b)	Std error	Wald Z value	Adjusted odds ratio (95% CI)	*p*-value
Age					
Early adulthood	−0.351	0.454	0.595	0.70 (0.29–1.72)	0.440
Middle-late adulthood	0				
Gender					
Male	−0.754	0.704	1.146	0.47 (0.12–1.87)	0.284
Female	0				
Marital status					
Single	−0.106	0.459	0.053	0.90 (0.37–2.21)	0.817
Married	0				
Education level	−0.005	0.495	< 0.001	0.99 (0.38–2.63)	0.992
Low	0				
Middle-high					
Working status	0.599	0.460	1.693	1.82 (0.74–4.49)	0.193
Have	0				
Non					
Income					
With	1.873	0.530	12.466	6.50 (2.30–18.39)	< 0.001*
Without	0				
Smoking					
Yes	0.366	0.612	0.359	1.44 (0.43–4.78)	0.549
No	0				
Took Alcohol					
Yes	−0.222	0.700	0.101	0.80 (0.20–3.16)	0.751
No	0				
Took drugs					
Yes	−1.122	0.549	4.174	0.33 (0.11–0.95)	0.041*
No	0				
Disease					
Have	−0.293	0.444	0.437	0.75 (0.32–1.78)	0.508
No	0				
Disability					
Have	−0.668	1.044	0.410	0.51 (0.07–3.96)	0.522
No	0				

* Significant at *p* < 0.05

## Discussion

It is found that majority of the homeless people in Kuala Lumpur were male, in their middle age and more than half of them were still single. These findings were consistent with findings by Pusat Transit Gelandangan Kuala Lumpur, Mohamad et al., and Muhammad Syafiq and Doris [3, 12, 17]. However, according to the Department of Social Welfare 2015 statistical report regarding homeless in Malaysia, 68.2% were female and majority of them were at 30–60 years old [18]. However, in America, the homeless population were at a younger age of 18–40 years old [19, 20]. Similar findings were found by other studies in which more than half of the homeless were single [12, 17, 21]. Marriage is not their priority as they can hardly afford to spend money even for themselves.

This study also found that majority of homeless people managed to complete their form five study only. Less than half of these homeless respondents were working. According to Mohamad et al. and Muhammad Syafiq and Doris, not all homeless people was jobless [12, 17]. However, this study also found that their income was relatively very low, which made it difficult for them to rent even a room in Kuala Lumpur. From this study, half of the homeless respondents had low income, i.e. just below the Malaysia’s average national poverty line household monthly income [22]. In view of their low education level, it is also difficult for them to get a good job. As mentioned earlier, Shelton et al., stated that their low education level was due to several risk factors such as being school dropout or having financial problems to pursue their study [6]. Another possible reason on why they did not continue their study was because higher education level was expensive [23]. According to Muhammed Abdul Khalid, the liberalization in terms of fees in public universities would have an impact towards the poor [24]. This finding is supported by other study findings which stated that these homeless people earn a low income job. They also hold small-scaled jobs paid with a meagre salary, for example picking up stuff such as cans or tins, arranging files, taking care of parking lots, helping friends in bundle shops, being a security guards, retail shop assistants, masseurs and sweepers [17, 25]. In fact, many studies including this study found that some of them were not paid for the job that they were hired for [12, 17, 25]. According to Mohamad et al., there were some employers who take advantage against this group, by not paying them accordingly [12].

Majority of the homeless people in this study were smokers, which is higher than overall Malaysians, i.e. 22.8% adults (aged > 15) or 43% of men smoked [26]. Majority of them did not consume alcohol. This finding was similar to the data from the Department of Welfare (*Jabatan Kebajikan Masyarakat*) which found that only 0.5% of the homeless population were alcoholic [27].

More than half of the respondents in this study had diseases with half of them having chronic diseases. According to Emma Woolley there were at least 85% of homeless people reported having chronic health problems such as asthma, bronchitis, hypertension with diabetes and diabetes [28]. However, most of the previous studies conducted in Malaysia emphasized mental health over other illnesses. On the other hand, infectious disease such as HIV were also found among this homeless population. Almost one fifth of the respondents were found to be HIV positive. Referring to Global Aids Response Progress Report Malaysia 2015, 11.7 cases per 100,000 Malaysian populations were HIV positive [29]. This finding is supported by a number of previous studies. National Coalition for The Homeless stated that the prevalence of HIV among homeless people was between 3 and 20%, and people living with HIV / AIDS were at higher risk of becoming homeless [8]. National Coalition for the Homeless and Kidder et al. found in their studies that homeless population has three times risk in getting HIV disease compared to the general population [8, 30]. This is supported by Hwang’s findings, who mentioned that they had the potential to be exposed with infectious diseases such as hepatitis A, B and C, tuberculosis (TB) and HIV / AIDS because of their low body’s immunity, perhaps due to poor nutrition apart from risky lifestyle such as free sex activity [31]. In addition, many researchers found that there is a relationship between homeless population and people living in poverty with mental health problems [12, 32]. Mohd Iqbal Haqim discovered in their study on person with mental disabilities, that most youth who were involved in drug-induced cases were known to be vagabonds and homelessness [32].

Although the homeless population interviewed has diseases, only half of them utilized health services. Despite low cost health facilities charges by the Malaysia government, i.e. only RM1 for outpatient clinic fee and RM5 for specialist clinic fee, only one third of homeless people utilized government health clinics and hospitals. This result is different from findings from the National Health Mobidity Survey 2015, where 60.1% of their respondents chose to get health services from government facilities while 39.9% sought treatment from private facilities [28]. Kidder et al. also found that community clinics were the only clinics frequented by the homeless population [30]. Less than half of the homeless population spent their own money from their small income to finance their own health and less than half of them received support. These study findings showed that the homeless underutilized healthcare services and this may due to their inability to pay as well as their physical disabilities. Their inability to pay may not be just in terms of paying for the services but this include accessing the healthcare services itself [33].

Further findings show that there was no significant relationship between socio-demographic factors with health service financier. However, gender had an association when receiving aid for health services especially among females. This study shows that women received more aid compared to men. Kaye et al. reported that women and those in the older age group needed health care assistance in the long run [34]. However not many studies in the past studied between these two variables. Health service finance among the homeless population usually did not look into the socio-demographic background of the individual.

With regard to working status as a form of socio-economic factor, this study has found that working status had an association with health service financier. Both working and income status were found to have significant association with health service financier. Within the working respondents, they used their own income to finance their health services. This is because they have earnings to be used during emergencies in terms of financing their health services. This result also shows that among beggars, they received health service finances from other sources. This include from various non-government organizations (NGO), Department of Social Welfare and many other foundations. Kaye et al. reported that community assistant was very much related to respondents’ socio-economic factors [34].

Fazel et al. showed that morbidity and mortality rate were higher among the homeless people [35]. In this study, it was found that having disabilities and had history of taking drugs significantly associated with health services financier among homeless besides depending on their own income. A study conducted in United States reported that health care assistance for HIV patients was available for almost 99% of HIV patients including the homeless people [35]. Kidder also mentioned that community clinics were among the services provided for the homeless community including those who were affected by HIV [35]. Nevertheless, not all health factors among this population were able to attract health services financier. This is because a person’s behavior does not reflect his or her intention when giving any form of aid. Based on current experience, Malaysian show high empathy and concern towards one who fells sick. Many NGOs continue giving aids to homeless population [3]. Study done by Rafiza et al. showed that NGOs social interaction with the homeless population was at good level [36].

Face to face interview was conducted to overcome the language barrier when communicating with the homeless who may find it difficult to comprehend the content of the questionnaire. However, possible disadvantage of face to face interviews may occur taking into account that homeless were often shy and embarrassed when interviewed by others, due to their condition. This could contribute to study limitation. To reduce this limitation, rapport were established with the temporary sheltered community during charity work done with NGOs and interview were done in the temporary sheltered itself at their own places. There were also difficulties in finding references as not many studies had been conducted among the homeless community with regards to physical health. Apart from that many studies too had been focusing on mental health issues only.

## Conclusion

In conclusion, utilization of healthcare services among homeless in Malaysia is still low especially when they have diseases. With no or only very low income, homeless are not able to pay for their healthcare. Above and beyond, they also receive less health financial aid. It is shown that their low utilization of health care services are due to their low ability to pay for the health fee or charges and therefore reduced accessibility to the health facilities. The main factors that play a role as health services financier among homeless community are using their own income or receiving aids for health services. This study suggests that government may need to collaborate with certain companies to allocate certain numbers of jobs to this homeless community with specific agreements instead of offering jobs to foreigners.

It is recommended that, with the current contribution by NGOs and government agencies such as Ministry of Health, Ministry of Women, Family and Community Development and Ministry of Human Resources plays an important role in delivering consistent and regular healthcare services to ensure that the homeless people are not left behind. Perhaps, in line with country’s transition into better health financing scheme in achieving universal coverage, it is recommended that special welfare card which is similar to welfare card for the disable people will be given to the homeless community for them to easily utilize healthcare services. Overall, the study findings highlight the health related needs of homeless for policymakers to take necessary action in improving their everyday life.

## Abbreviations

AIDS: Acquired ImmunoDeficiency Syndrome
HIV: Human Immunodeficiency Virus
NGOs: Non-governmental organizations
OR: Odd Ratio
RM: Ringgit Malaysia
SD: Standard Deviation
UKM: Universiti Kebangsaan Malaysia
UNHCR: United Nations High Commissioner on Refugee
USA: United States of America
YKN: Yayasan Kebajikan Negara
